# Robotic Vascular Resection in Pancreatic Ductal Adenocarcinoma: A Systematic Review

**DOI:** 10.3390/jcm13072000

**Published:** 2024-03-29

**Authors:** Victoria Zecchin Ferrara, Alessandro Martinino, Francesco Toti, Davide Schilirò, Federico Pinto, Francesco Giovinazzo, on behalf of the SMAGEICS Group

**Affiliations:** 1 Faculty of Medicine and Surgery, University of Padua, 35122 Padua, Italy; victoria.zecchinferrara@studenti.unipd.it; 2 Department of Surgery, Duke University, Durham, NC 27708, USAdavide.schiliro@duke.edu (D.S.); 3 Department of Surgery, ASST Santi Paolo e Carlo, 20100 Milan, Italy; 4 Department of Surgery, University of Illinois at Chicago, Chicago, IL 60607, USA; 5 Department of Surgery, Fondazione Policlinico Universitario Agostino Gemelli IRCCS, 00131 Rome, Italy; 6 Department of Health Sciences, UniCamillus-Saint Camillus International University, 00131 Rome, Italy; 7 Department of Surgery, Saint Camillus Hospital, 31100 Treviso, Italy

**Keywords:** pancreas, robotic surgery, resection, vascular, ductal adenocarcinoma

## Abstract

(1) **Background:** This study comprehensively compared robotic pancreatic surgery with vascular resection (RPS-VR) to other surgical procedures in the treatment of pancreatic ductal adenocarcinoma (PDAC). (2) **Methods:** A systematic review of relevant literature was conducted to assess a range of crucial surgical and oncological outcomes. (3) **Results:** Findings indicate that robotic surgery with vascular resections (VRs) significantly prolongs the duration of surgery compared to other surgical procedures, and they notably demonstrate an equal hospital stay. While some studies reported a lower conversion rate and a higher rate of blood loss and blood transfusion in the RPS-VR group, others found no significant disparity. Furthermore, RPS-VR consistently correlated with comparable recurrence rates, free margins R0, postoperative mortality, and complication rates. Concerning the last one, certain reviews reported a higher rate of major complications. Overall survival and disease-free survival remained comparable between the RPS-VR and other surgical techniques in treating PDAC. (4) **Conclusions:** The analysis emphasizes how RPS-VR is a resembling approach in terms of surgical outcomes and aligns with existing literature findings in this field.

## 1. Introduction

Pancreatic ductal adenocarcinoma (PDAC) is a subtype of pancreatic cancer known for its late-stage diagnosis, aggressive nature, and limited treatment options. The 5-year survival rate is approximately 5% [1]. In addition, 20% of pancreatic cancer patients have localized disease that is resectable, and the remaining patients are diagnosed with tumors involving the superior mesenteric artery (SMA), superior mesenteric-portal vein (SMV-PV), or celiac axis (CA) [2], which are either locally progressed/borderline resectable (30%) or blatantly metastatic diseases (50%) [3,4]. To maximize the likelihood of a radical resection (R0) in these situations, a pancreaticoduodenectomy (PD) with en-bloc resection of the affected venous segment is typically scheduled following neoadjuvant therapies [2,5]. As a leading cause of cancer-related deaths globally, PDAC demands increased research and innovative approaches for both early diagnosis and effective therapeutic interventions. Surgical resection has provided the only chance for a cure in patients with PDAC, but the 5-year survival rate is still low (20%) in patients with margin-negative resection [6]. Conventional open surgery involves a large abdominal incision and direct manual manipulation by the surgeon. While effective, it entails considerable morbidity and protracted recovery periods. In contrast, minimally invasive surgery has been utilized progressively as it enables internal organ visualization and manipulation through small incisions. This technique reduces tissue trauma, pain, and postoperative recovery time. When addressing pancreatic cancer with vascular involvement, the juxtaposition of these surgical modalities becomes crucial in the pursuit of improving postoperative recovery, complication rates, and long-term outcomes. PD-VR is a more complicated procedure than pancreatoduodenectomy alone [7,8,9,10]. As a result, the traditional open technique, long regarded as the gold standard, has been used for the majority of this procedure [11,12]. A few specialized centers have reported minor PD-VR series performed using a minimally invasive technique in recent years [13,14,15,16]. With the improvement of minimally invasive techniques, LDP and even LPD have been used to treat PDAC with effectiveness [17]. Despite the potential benefits, the adoption of robotic surgery for treating pancreatic ductal adenocarcinoma (PDAC) is still in the initial phase. Data regarding the feasibility of robotic pancreatoduodenectomy (RPD) with vascular resection (VR) are only available in limited reports from high-volume centers [13,16,18,19,20]. Ongoing research is dedicated to unraveling long-term oncological outcomes, refining patient selection criteria, and optimizing techniques for complex cases of these procedures. With this systematic review, we aim to contribute valuable insights to the ongoing discourse surrounding the safety and feasibility of robotic pancreatic surgery with vascular resection.

### Historical Development

Pancreaticoduodenectomy (PD) has been widely applied since Allen Whipple introduced it in 1940. Although the procedure has evolved, it remains basically similar to what is performed today [21]. Tumors of the body and pancreatic tail are treated with distal pancreatectomy (DP) and splenectomy with corresponding lymphadenectomy from the left side of the vascular systems.

Globally, minimally invasive pancreatic surgery has gained popularity as a result of technological advancements. The first laparoscopic pancreatectomy was described in 1996, while the first robotic pancreatic resection was reported in 2003 [22,23]. Open, laparoscopic, and robotic pancreatic surgery all have advantages for treating patients with pancreatic disorders, according to a recent international evidence-based guideline on minimally invasive pancreatic surgery. Promising clinical outcomes can be achieved by applying these advanced technologies [24].

Minimally invasive pancreatic surgery (MIPS) has been slower to adopt compared to other minimally invasive abdominal procedures due to the challenging position of the pancreas within the abdomen, its proximity to important vessels, and the necessity for intricate reconstruction. RPD is more costly than LPD; nevertheless, it offers surgeons three-dimensional stereoscopic views of the surgical field, enhanced dexterity, tremor filtration, and the capability to perform complex dissections, sutures, and knots with unprecedented precision [25]. However, its complexity makes it technically demanding: RPD-VR needs to be performed by surgeons who have surpassed their learning curve.

The optimum course of treatment is radical surgical resection; however, the existence of major vessel invasion is typically taken into consideration as an indicator of unresectability. A tumor involving the arterial and/or the portal/mesenteric axis is classified as borderline resectable pancreatic cancer (BRPC) in accordance with standards published by the National Comprehensive Cancer Network (NCCN) [26]. However, a recent international consensus statement [27] on the definition of borderline resectable pancreatic cancer (BRPC) has taken into account not only the anatomic relationship between the tumor and vessels but also biological and conditional dimensions such as the patient’s performance status and suspicion for distant or lymph node metastases. Vascular resection and reconstruction can be accomplished through a variety of techniques depending on the vessels involved and the extent to which the tumor has extended into the vessel wall. These techniques include partial resection with direct closure of the defect, segmental resection with end-to-end anastomosis, segmental resection with an artificial or venous graft interposed, and resection and reconstruction of multiple vessels [28].

The first surgical procedure focusing on the superior mesenteric vein (SMV) was described by Moore in 1951 during pancreatic surgery. Since then, venous resections have been improved and changed to become a standard surgical approach for all kinds of pancreatic surgeries in patients with PDAC, such as pancreaticoduodenectomy and distal or total pancreatectomies.

Venous (superior mesenteric vein, portal vein) infiltration occurs more frequently than arterial (hepatic artery, celiac trunk, superior mesenteric artery) invasion. When performing an extended gastrectomy that included a distal pancreatectomy, Appleby first described arterial resection during abdominal surgery in the 1950s [29].

## 2. Material and Methods

In this systematic review, a comprehensive search and analysis of various research concerning robotic surgery with vascular resections for pancreatic ductal adenocarcinoma (PDAC) was conducted. An AMSTAR 2 checklist is included as Supplementary Materials File S1 to facilitate the assessment of the presented systematic review [30].

### 2.1. Objectives and PICO Process

The primary objective of this systematic review is the assessment of postoperative mortality and overall/disease-free survival in the different analyzed groups. The secondary objectives encompass assessing parameters such as blood loss, blood transfusion, duration of surgery, complication rate, conversion rate, hospitalization time, surgical margins R0, and recurrence. As functional outcomes, parameters such as postoperative pancreatic fistula (POPF) and delayed gastric emptying (DGE) were evaluated.

Utilizing the PICO criteria in framing a research question, this study aimed to investigate the following: “In patients undergoing surgical treatment with vascular resection for PADC (P), does robotic surgery (I) compare to open and laparoscopic surgery (C), result in differences in postoperative mortality, overall/disease-free survival, blood loss, blood transfusion requirements, duration of surgery, complication rate, conversion rate, hospitalization time, surgical margins, recurrence? (O) [31]”.

### 2.2. Search Strategy

The systematic review adhered to the guidelines outlined in the PRISMA statement for the conduct and reporting of data [32]. The research focused on articles published in English in peer-reviewed journals. Studies were identified by searching multiple literature databases, including Scopus, PubMed, and Embase. Employing an advanced search strategy, we employed terms such as “robotic” AND “pancreas” AND “vascular resection”. Results were admitted from the time of inception up to and including December 2023. Moreover, manual screenings of reference lists from pertinent articles were conducted, aiming to identify further relevant studies.

### 2.3. Inclusion and Exclusion Criteria

Randomized controlled trials, meta-analyses, systematic reviews, case-matched studies, and retrospective cohort studies were all eligible for inclusion if they were focusing on patients with pancreatic ductal adenocarcinoma (PDAC). The selected articles were required to analyze robotic pancreatic surgery with vascular resections versus other techniques of pancreatic surgery. We excluded all non-English language and no full-text available articles. We also excluded articles about minimally invasive surgery (MIS) in which robotic surgery was not specifically considered.

### 2.4. Data Extraction

At least two reviewers (VZF, FP) independently collected all data, ensuring meticulous data collection by extracting diverse outcomes. Data were gathered using customized tables containing the following information: study design, study duration, year of publication, sample size, number of patients enrolled in each study (including mean age and gender), primary and secondary outcomes, functional outcomes, and techniques used for vascular resection. In instances of multiple publications from the same working group, only the most recent or informative publication was considered to prevent duplication of data.

## 3. Results

An extensive search retrieved a total of 223 records. During the initial screening phase, 100 articles were excluded due to duplication, and 95 were excluded for not meeting the inclusion criteria. Consequently, only 28 articles remained eligible for a thorough full-text review.

**Figure 1:**
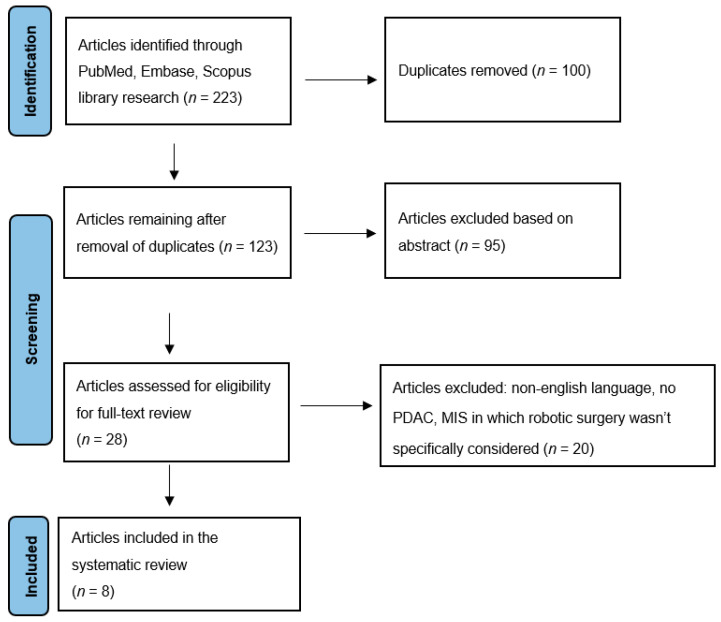
PRISMA flow diagram.

Ultimately, our study comprised eight articles, consisting of six retrospective reviews, one systematic review, and one case report. See Figure 1.

Among them, six articles focused on robotic pancreatoduodenectomy with VR [13,16,33,34,35,36], while two articles addressed robotic pancreatectomy with VR [37,38]. The selected articles analyze the differences in robotic pancreatic surgery with vascular resections compared to the following techniques of pancreatic surgery: three articles compare it to RPD with no VR [13,16,34], two articles compare it to OPD with no VR [35,37], one article compares it to OPD with VR [33], and one article compares it to LPD with no VR [38]. 

Table 1 is presented below [13,16,33,34,35,36,37,38].

### 3.1. Blood Loss

The analysis of blood loss revealed a significant difference between the robotic pancreatic surgery with the VR group and the RPD with no VR group [16,34]. However, three studies found no significant difference when comparing RPS-VR to RPD [13], OPD-VR [33], and LPD [38].

These findings show that larger studies with robotic surgery are required to demonstrate a significant reduction of intraoperative blood loss, which could be useful in minimizing the risk of complications and ensuring patient safety.

### 3.2. Blood Transfusion

When assessing the need for blood transfusion, two reviews [16,34] reported a higher rate in the RPD-VR group compared to RPD with no VR group. However, one study [13] found no significant difference between the two groups, suggesting that the effectiveness of RPD-VR in reducing blood transfusions may depend on specific patient characteristics or procedural factors, such as patient fitness, the presence of comorbidities, or the surgeon’s experience and used technique. Regarding the comparison with LPD [38], no significant difference was found.

### 3.3. Duration of Surgery

The analysis of the duration of surgery across four articles revealed a significant difference between the robotic pancreatic surgery with the VR group and the robotic or laparoscopic surgery without the VR group [13,16,34,38]. The finding suggests that patients undergoing RPD-VR are more likely to experience an extended duration of the procedure. However, one retrospective review [33] did not find a significant difference when comparing RPD-VR to OPD-VR, indicating that other factors may influence the length of surgery.

### 3.4. Complication Rate

Among the studies that evaluated the overall complication rate, five reviews found no significant difference between the RPS-VR group compared to other pancreatic surgical procedures groups [13,16,33,34,38]. However, one included study found a significant difference in terms of major complications (complications graded ≥ III were considered severe) when comparing robotic pancreatic surgery with VR to RPD [34].

### 3.5. Conversion Rate

Regarding the conversion rate, three articles were included in the analysis [13,34,38]. Two studies found no significant difference in the RPD-VR group compared to RPD with no VR group [13,34]. However, one retrospective review found a lower conversion rate when comparing RPD-VR to LPD [38].

### 3.6. Hospitalization Time

The analysis of hospitalization time in patients who underwent RPD-VR revealed that four reviews detected a comparable hospital stay when considering the RPD group [13,16,34] or the OPD-VR group [33]. However, one study [38] found a longer hospitalization time when comparing RDP-VR to the LPD group.

### 3.7. Postoperative Mortality

Among the included articles, five reported no significant difference in mortality rates between the robotic surgery group with VR and other pancreatic surgical technique groups [13,16,33,34,38].

### 3.8. Surgical Margins R0

Among the research that evaluated this oncologic outcome, four reported no significant difference in rates of surgical margins R0 in the RPS-VR group [13,16,34,38]. One retrospective review [33] found a lower incidence of R1 resection in the RPD-VR cohort; however, it was not significantly different compared to the open approach with VR (7.1% versus 12.9%, *p* = 1.000).

### 3.9. Recurrence

Regarding cancer recurrence, three articles were included in the analysis [33,34,38]. These did not detect any significant difference compared to other pancreatic surgical techniques, implying that RPS-VR is a resembling approach in terms of controlling cancer recurrence.

### 3.10. Overall Survival and Disease-Free Survival

Data from three studies [33,34,38] showed no significant difference regarding overall survival and disease-free survival when comparing RPS-VR to other pancreatic surgical techniques.

## 4. Discussion

In the medical world, there is broad agreement that robotic pancreatic surgery with VR is practical, safe, and advantageous over open procedures. However, it is quite complicated and surgeons need extensive training to master the skills required.

The results of this review’s comprehensive analysis shed light on the comparative outcomes of robotic surgery in the context of PDAC. Findings provide valuable insights into the advantages and limitations of these surgical approaches, contributing to the ongoing research about the optimal treatment strategy for this challenging condition. We have been using PD-VR in open surgery since the late 1980s [39]. Since then, our efforts were finalized to maximize local radicality and to establish standardized techniques permitting the safe handling and reconstruction of large peripancreatic vessels. Nowadays, it is considered a practical and conventional option for various pancreatic surgeries, including PDAC.

These operations are usually performed through an open approach that allows straightforward vascular reconstruction, which is frequently achieved by end-to-end anastomosis.

Other forms of vein reconstruction and resection are possible in MIPD, although they come with more difficulty during the procedure. While direct restoration of the superior mesenteric/portal vein (SMV/PV) is assisted by either intestine or hepatic mobilization, the splenic vein can be easily anastomosed to the left renal vein in OPD [40]. Vein reconstruction in MIPD introduces additional complexity for various reasons:

- the bowel cannot be fully mobilized and pushed towards the liver;
- if the surgeon uses a jump graft, he or she must take into consideration that at the conclusion of the procedure the reverse Trendelenburg position will be abolished, which could create vascular kinking if the graft is too long;
- vascular control can be problematic when the SMV is involved near the mesenteric root: it could be quite challenging to reposition the clamp on the SMV after the vein has been separated;
- if the vein segment to be resected includes the splenomesenteric junction, managing the splenic vein presents additional challenges;
- in the event of uncontrolled bleeding, an emergency conversion to open surgery is required.

The potential of a safe outcome determines whether vein resection should be performed or if conversion to open surgery should be used instead. Conversion to open surgery is necessary if the surgeon is uncomfortable performing RPD-VR.

The group of study of E.F Kauffman et al. [41] provided the tips and tricks included in 36 RPD-VR: while some were obtained from OPD, others were imported from their robotic experience with pancreatic and kidney transplantation. Each type of vein resection (four types according to the ISGPS) was described in detail and shown in a video. There was only one conversion to open surgery (2.8%) due to widespread bleeding in a patient who had numerous comorbidities and a prior bone marrow transplant. The feasibility learning curve was completed after six procedures; after this turning point, 90-day mortality declined: the overall 90-day mortality was 8.3%.

The International Study Group of Pancreatic Surgery (ISGPS) has classified the different types of venous resection and reconstruction [42]:

- In the case of marginal (<25%) circumferential involvement, a small side-wall resection followed by a primary direct venorraphy (Type 1) is performed.
- If <50% of the vein circumference is involved, a larger side-wall resection followed by a patch venorraphy (Type 2) is carried out.
- If a venous involvement > 50% is detected, and the length of the vascular defect is less than 3 cm, a segmental resection with direct repair is performed (Type 3).
- If an encasement of >180° is detected, and the length of post- resection venous defect is more than 3 cm, an interposition graft is used for vascular repair (Type 4).

Overall, type 3 anastomosis is most commonly utilized [33,34,35], and type 4 resection/reconstruction is associated with the longest operative time and highest estimated blood loss.

As in OPD, a superior mesenteric artery (SMA) first approach is usually employed to release the head of the pancreas, ultimately leaving the specimen only attached to the involved venous segment. Information on arterial anatomy is used to establish the most convenient route for the artery first approach to the SMA and to reduce the possibility of iatrogenic vascular injury, especially in case of variations in arterial liver supply. The entire surgical team should be aware of this occurrence and have a rescue strategy in case of iatrogenic arterial injury. When distant metastases are not found, highly selected patients may undergo vascular resections following neoadjuvant chemotherapy [26]. The majority of surgeons prefer an open technique when performing planned arterial resection; the incidence of unplanned conversion in minimally invasive pancreatoduodenectomy surpasses 40% when vascular resection is necessary, which serves as indirect evidence in favor of this preference [43]. Patients with minimal arterial involvement that is unintentionally discovered during surgery are given an arterial resection if a robotic technique proves to be feasible. Though the standard course of action for patients with preoperative signs of vascular involvement is to undergo neoadjuvant chemotherapy, the choice for upfront resection should be considered a rare exception, determined by unanticipated intraoperative discoveries.

Massive bleeding, requiring emergent conversion, can happen and is associated with a mortality rate as high as 60% [44].

Patient selection for RPD and potential VR was based on anatomic considerations identified in the preoperative imaging (CT, MRI, EUS). When selecting patients for RPD, as more experience was gained, four studies [13,16,33,35] progressively modified their selection criteria. Indeed, some patients were initially not included in the results; however, surgeons gradually used RPD for more difficult cases with broader involvement in imaging.

Common exclusion criteria were as follows: patient denial of robotic approach, unavailability of the robotic system, general unsuitability for minimally invasive techniques, intraperitoneal or extraperitoneal metastases, major vein involvement (>270°, segmental occlusion or thrombosis), and patients with high BMI (≥35 kg/m^2^ were initially excluded). Additional radiological or endoscopic investigations were usually considered if the patient had central obesity with a body max index ≥ 35 kg/m2 and was deemed either borderline resectable or locally advanced. Initially, VR was limited to short-segment abutments (<90-degree abutment over a 1–2 cm segment) of the SMV/PV. As the experience matured, surgeons used the robotic approach firstly for limited vein involvement (i.e., unilateral contact, <180°, without distortion of vessel contour) and after for more challenging cases on patients with more extensive involvement in imaging (venous encasement > 180° or 2 cm segment). Patients with longer segment involvement (>2 cm) or more progressed vascular encasement were reserved for an open approach. While modest artery involvement was carefully evaluated on a case-by-case basis, overall arterial encasement was regarded as an absolute contraindication.

Considering the complexities involved in obtaining the best possible oncologic outcomes, the decision between robotic surgery and open or laparoscopic procedures should be made with consideration for the unique features of the tumor as well as the subtleties of the anatomical context. The group of study of J.D. Beane et al. [13] reported the outcomes and learning curve of RPD-VR: their findings suggest that pancreatic surgeons who have previously mastered their learning curves can safely expand the RPD selection criterion to encompass major vascular resection. In summary, RPD-VR is a safe and practical treatment that may be carried out in a limited number of patients by surgeons who have experience with RPD: surgeons who have completed more than 80 RPDs can see improvements in operative time for RPD-VR in as few as 35 cases.

Consequently, high-volume centers that have already outgrown their learning curve with the conventional process should proceed cautiously when implementing RPD-VR [45,46].

This review may encounter limitations regarding the quality of the primary research studies. However, rigorous inclusion criteria were applied to ensure the reliability of the selected studies.

## 5. Conclusions

In conclusion, this analysis indicates that the majority of the studies found in the literature consider robotic pancreatic surgery with VR a feasible approach, but at the price of increased operative difficulty. Moreover, the comparable complication rates, postoperative mortality, overall survival, recurrence, and surgical free margins R0 associated with RPS-VR indicate an overall safety profile comparable to other surgical procedures. These practical implications are particularly relevant when dealing with a PDAC with vascular infiltration. The observed higher estimated blood loss, transfusion rates, and the longer surgical time should be considered in the context of personalized treatment strategies, where patient-specific factors and tumor characteristics play a crucial role in decision-making.

To improve our understanding of robotic vascular resection in PDAC, there is a compelling need for more information to draw final conclusions on safety. We do not, however, advise groups that are inexperienced with PD-VR or that have not yet completed the RPD learning curve to undertake these more complex procedures. Larger studies involving neoadjuvant chemotherapy are also needed to confirm any potential benefits for PDAC; these studies may increase survival among PDAC patients with VR undergoing both RPD and OPD.

## Figures and Tables

**Table 1:** Features of articles included. RPD: robotic pancreatoduodenectomy; LPD: laparoscopic pancreatoduodenectomy.

Author et al.	Year	Type of Study	Number of Patients
Beane J.D. et al. [13]	2019	Retroprospective review	° 380 RPD-VR: 50, RPD: 330
Jin J. et al. [33]	2022	Retroprospective review	° 84 RPD-VR: 14, OPD-VR: 70
Kauffman E. F. et al. [16]	2016	systematic review + literature review	° 130 RPD-VR: 14, RPD: 116
Kauffman E. F. et al. [37]	2020	Retroprospective review	° 361 RAPR-VR: 31, RAPR:330
Marino M. V. et al. [34]	2020	Retroprospective review	° 83 RPD-VR: 10, RPD: 73
Kauffman E. F. et al. [35]	2020	Retroprospective review	° 184 RPD-VR: 22, RPD: 162
Machado M. A. C. et al. [36]	2021	Case report	
Jeffrey W. C. et al. [38]	2023	Retroprospective review	° 542 RPD: 103, LPD: 439

OPD: open pancreatoduodenectomy; RAPR: robot-assisted pancreatic resection.

## Supplementary Materials

The following supporting information can be downloaded at: https://www.mdpi.com/article/10.3390/jcm13072000/s1, File S1: AMSTAR 2 checklist.

## Abbreviations

PDAC	Pancreatic ductal adenocarcinoma
RPS-VR	Robotic pancreatic surgery with vascular resection
RPD	Robotic pancreatoduodenectomy
LPD	Laparoscopic pancreatoduodenectomy
LDP	Laparoscopic distal pancreatectomy
OPD	Open pancreatoduodenectomy
VR	Vascular resection
DP	Distal pancreatectomy
RAPR	Robot-assisted pancreatic resection
